# Genetic signature of differentiated thyroid carcinoma susceptibility: a machine learning approach

**DOI:** 10.1530/ETJ-22-0058

**Published:** 2022-08-17

**Authors:** Giulia Brigante, Clara Lazzaretti, Elia Paradiso, Federico Nuzzo, Martina Sitti, Frank Tüttelmann, Gabriele Moretti, Roberto Silvestri, Federica Gemignani, Asta Försti, Kari Hemminki, Rossella Elisei, Cristina Romei, Eric Adriano Zizzi, Marco Agostino Deriu, Manuela Simoni, Stefano Landi, Livio Casarini

**Affiliations:** 1 Unit of Endocrinology, Department of Biomedical, Metabolic and Neural Sciences, University of Modena and Reggio Emilia, Modena, Italy; 2 Unit of Endocrinology, Department of Medical Specialties, Azienda Ospedaliero-Universitaria, Modena, Italy; 3 Institute of Reproductive Genetics, University of Münster, Münster, Germany; 4 Department of Biology, University of Pisa, Pisa, Italy; 5 Hopp Children’s Cancer Center (KiTZ), Heidelberg, Germany; 6 Division of Pediatric Neurooncology, German Cancer Research Center (DKFZ), German Cancer Consortium (DKTK), Heidelberg, Germany; 7 Biomedical Center, Faculty of Medicine and Biomedical Center in Pilsen, Charles University in Prague, Pilsen, Czech Republic; 8 Division of Cancer Epidemiology, German Cancer Research Center (DKFZ), Heidelberg, Germany; 9 Department of Endocrinology, University Hospital, Pisa, Italy; 10 Polito^BIO^ Med Lab, Department of Mechanical and Aerospace Engineering, Politecnico di Torino, Italy; 11 Center for Genomic Research, University of Modena and Reggio Emilia, Modena, Italy

**Keywords:** differentiated thyroid cancer, machine learning, single nucleotide polymorphism

## Abstract

To identify a peculiar genetic combination predisposing to differentiated thyroid carcinoma (DTC), we selected a set of single nucleotide polymorphisms (SNPs) associated with DTC risk, considering polygenic risk score (PRS), Bayesian statistics and a machine learning (ML) classifier to describe cases and controls in three different datasets. Dataset 1 (649 DTC, 431 controls) has been previously genotyped in a genome-wide association study (GWAS) on Italian DTC. Dataset 2 (234 DTC, 101 controls) and dataset 3 (404 DTC, 392 controls) were genotyped. Associations of 171 SNPs reported to predispose to DTC in candidate studies were extracted from the GWAS of dataset 1, followed by replication of SNPs associated with DTC risk (*P* < 0.05) in dataset 2. The reliability of the identified SNPs was confirmed by PRS and Bayesian statistics after merging the three datasets. SNPs were used to describe the case/control state of individuals by ML classifier. Starting from 171 SNPs associated with DTC, 15 were positive in both datasets 1 and 2. Using these markers, PRS revealed that individuals in the fifth quintile had a seven-fold increased risk of DTC than those in the first. Bayesian inference confirmed that the selected 15 SNPs differentiate cases from controls. Results were corroborated by ML, finding a maximum AUC of about 0.7. A restricted selection of only 15 DTC-associated SNPs is able to describe the inner genetic structure of Italian individuals, and ML allows a fair prediction of case or control status based solely on the individual genetic background.

## Introduction

Thyroid cancer is the most common endocrine neoplasia with a worldwide estimated age-standardized incidence rate of 6.7 per 100,000 in 2018 (1). Differentiated thyroid carcinoma (DTC) is the most frequent subtype of thyroid cancer with increasing incidence in the last 20 years, likely because of the increased knowledge of associated risk factors and ameliorated diagnostic procedures (2). However, most DTC have a favourable prognosis (3), and the diagnostic-therapeutic procedures should aim to avoid both delayed diagnosis and overmedication.

To date, the management of thyroid nodules suspected to be DTC is mainly guided by the sonographic risk pattern and the coexistence of other risk factors (3). Genetics could play a role in helping the diagnostic process, assuming the possibility to stratify patients according to a personalized risk profile (4). This stems from the observation that blood relatives of patients diagnosed with DTC show a highly increased risk for the disease, implying the existence of an important genetic component (5, 6). The role of genes in the aetiology of DTC has been studied in populations and most of the risk alleles have been identified by case/control and genome-wide association studies (GWAS) (7, 8, 9, 10, 11, 12, 13, 14, 15). However, it is still difficult to predict the individual risk of DTC based on the existing data, likely because of a complex interaction among multiple co-inherited low/moderate penetrant alleles. In fact, one single common variant *per se* is weakly associated with increased DTC risk, which could instead emerge as a cumulative effect of several single nucleotide polymorphisms (SNPs) with individual low impact. Thus, the overall risk could be the result of complex gene–gene and gene–environment interactions.

In order to take into account multiple alleles, the measure of disease susceptibility could be provided by calculating the polygenic risk score (PRS), where each variant allele is treated as an individual, independent, risk factor, and subjects are stratified according to the number of risk alleles, in additive or weighted models. The so calculated cancer risk may achieve relatively high odd ratio (OR) values (16, 17, 18, 19, 20, 21, 22). For DTC, it has been shown that people carrying ≥14 risk alleles have an about 8-fold increased risk compared to people carrying ≤7 risk alleles (23, 24). Therefore, the PRS is a promising method for risk prediction. However, gene–gene interactions are likely too complex to be explained by simple additive or weighted models and alternative methods are under exploration.

Machine learning (ML) is increasingly used for predicting individuals’ inherited genomic susceptibility to cancer (25). Another interesting approach is represented by Bayesian statistics for population genetics, in which individuals are assigned to ethnic subgroups or phenotypes according to their underlying genetic structure (26, 27). Genetic data may serve to run ML diagnostic analyses aimed at stratifying individuals into disease risk categories (28). However, these methods have not been fully exploited for dissecting complex traits, such as the susceptibility to cancer, assuming it as phenotype information. To the best of our knowledge, ML has never been applied before to the study of genetic predisposition to DTC.

In this replication study, we aimed to assess the genetic signatures associated with the predisposition to DTC. For this purpose, a small number of SNPs descriptive of a DTC-related genotype were selected in three independent genetic datasets and confirmed by Bayesian statistics. The diagnostic performance of the selected markers in categorizing the case/control state of subjects was evaluated by ML techniques.

## Methods

### Study design

**Figure 1:**
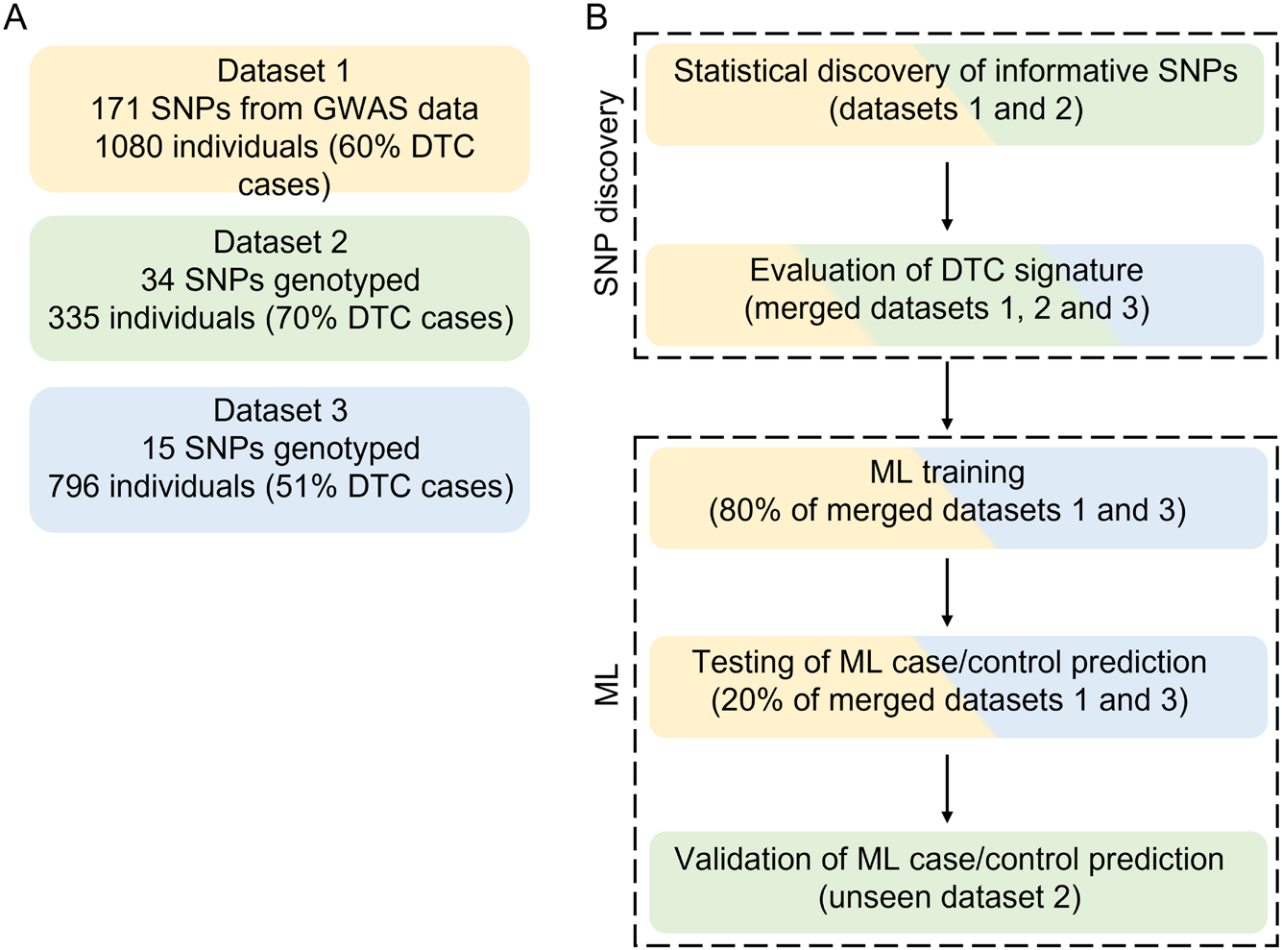
Datasets and project’s pipeline. (A) Summary of dataset composition, highlighting the progressive refinement of the SNP selection process. Dataset 1 SNPs were extracted from a GWAS (12), while datasets 2 and 3 SNPs were genotyped *ad hoc* for potentially informative SNPs. The 34 SNPs significantly associated with DTC in dataset 1 were genotyped *ad hoc* and checked for relevance in the independent dataset 2. Then, 15 SNPs highly associated with DTC in both datasets 1 and 2 were further genotyped *ad hoc* in the independent dataset 3. (B) Procedure for statistical SNP discovery and subsequent ML implementation. After SNPs selection, we tested the capability of the 15 selected SNPs to provide a DTC genetic signature in the merged datasets 1, 2 and 3 with Bayesian statistics for population genetics. Then, ML methods were run to confirm the case/control state of individuals using the selected 15 SNPs as input variables. An extended dataset was built by merging the two largest datasets (1 and 3), to obtain a pool of randomly chosen ‘training’ (80% of the merged dataset) and ‘testing data’ (20%). After finding the most effective ML algorithms, a validation analysis was set on the dataset 2. Shaded colours highlight involved datasets. Yellow = dataset 1; green = dataset 2; light-blue = dataset 3.

Briefly, we identified a relatively low number of SNPs highly associated with DTC by sequential association analyses in three independent case/control series. These SNPs served as genetic information to describe the case/control status of Italian individuals by ML methods. First, a selection of 171 candidate DTC-associated SNPs was obtained from the literature (see paragraph ‘SNPs selection’). The SNP list was further reduced after testing for SNPs association with the disease. For this purpose, genetic data from two of the available datasets (datasets 1 and 2; Fig. 1A), each comprising Italian DTC subjects and healthy controls, were used. Briefly, 34 SNPs considered significantly associated with DTC in dataset 1 were genotyped *ad hoc* and checked for relevance in the independent dataset 2. SNP selection criteria are reported in detail in the section ‘Statistical analysis’. Finally, a total of 15 SNPs highly associated with DTC in both datasets were obtained and further genotyped *ad hoc* in the independent dataset 3. Their potential of describing DTC signature was confirmed by a control Bayesian clustering in the merged three datasets (see section ‘Bayesian statistics for population genetics’). ML methods were run to confirm the case/control state of individuals using the selected 15 SNPs as input variables. For this purpose, an extended dataset was built by merging the two largest datasets (1 and 3) to obtain a pool of randomly chosen ‘training’ (80% of the merged dataset) and ‘testing data’ (20%). After finding the most effective ML algorithms, a replication analysis was set on the dataset 2. The whole procedure is summarized in Fig. 1B.

### Subjects

Dataset 1 has been previously described in a GWAS on DTC (12). It included Italian DTC cases and controls recruited consecutively from the Department of Endocrinology, University Hospital of Pisa, Italy, in the period January 2009–August 2011 (12). Overall, the genotypes of 649 DTC patients and 431 healthy controls were considered. Dataset 2 included 234 Italian DTC patients and 101 healthy controls recruited at the Unit of Endocrinology, University Hospital of Modena, Italy, between 2008 and 2012. These individuals were genotyped for 34 DTC-associated SNPs (‘SNP selection’ section) after DNA extraction from blood samples (Supplementary text, see section on supplementary materials given at the end of this article). Dataset 3 included 404 DTC subjects and 392 controls recruited at the Department of Endocrinology, University Hospital of Pisa, Italy, between September 2011 and December 2012, and subjected to genotyping for 15 DTC-associated SNPs (‘SNP selection’ section) after extraction of DNA from blood. All the DTC diagnoses have been histologically confirmed after thyroidectomy. Controls were recruited among healthy volunteers without known thyroid disease and/or with negative thyroid ultrasound. In details, controls of datasets 1 and 3 comprised healthy individuals without known thyroid disease recruited during a routine health screening or blood donor volunteers. Controls of dataset 2 were volunteers recruited by local advertisement as the control group for an ongoing case/control study on thyroid cancer; one of the participants had a personal history of thyroid disease and they had never undergone any thyroid ultrasound scan before; they performed thyroid ultrasound and thyroid resulted to be normal for size, position and echogenicity, without cystic or nodular lesions. All the subjects enrolled in the three independent datasets were unrelated.

Information about sex, age at diagnosis of DTC for cases and age at recruitment for controls and anthropometric measurements (height and weight) were collected. BMI was also calculated as the weight (kg)/height (m)^2^ ratio. Individuals underwent peripheral blood withdrawn and samples were stored at −20°C until analysis. DNA was extracted from EDTA-venous blood samples using standard methodologies. In dataset 1, SNPs missing in the GWAS were obtained by imputation by exploiting the linkage disequilibrium (LD) blocks (29). SNP genotyping in datasets 2 and 3 was performed with the iPLEX® assay (Life & Brain GmbH, Bonn, Germany) (Supplementary text).

The local Ethics Committees of Modena and Pisa (Italy) approved the study (Protocol Nr. 122/08, Nr. 7116/09 and Nr. 2359/14), and all participants signed a written informed consent.

### SNP selection

**Figure 2:**
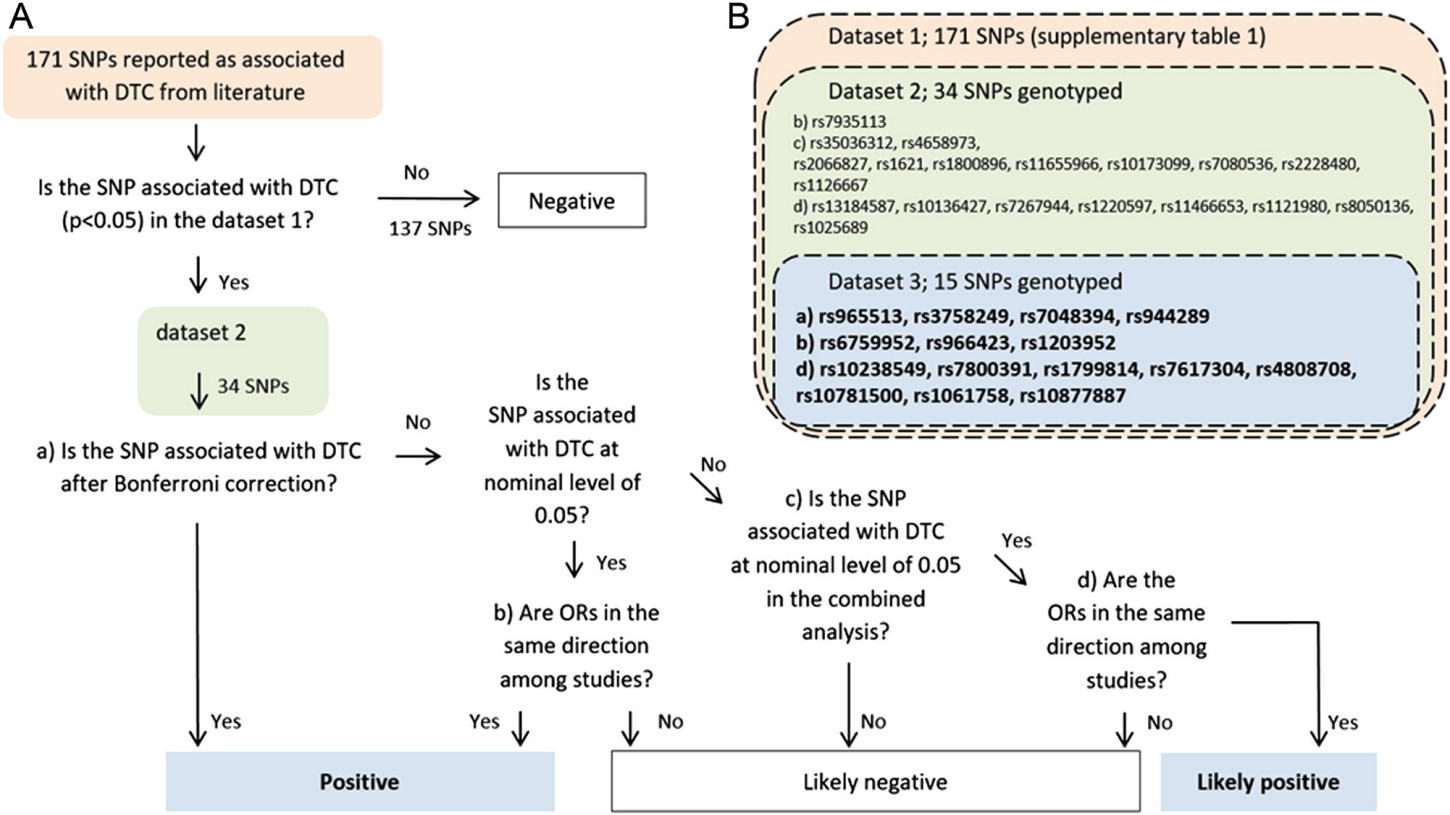
SNP selection. (A) Criteria used for SNP selection. (B) SNP subsets. Among the 171 SNPs selected from the literature (Supplementary Table 1), only 34 were associated with DTC in dataset 1 (*P* < 0.05; Supplementary Table 2) and genotyped in dataset 2. Fifteen SNPs were finally selected from dataset 2 as variables for ML analysis and genotyped in dataset 3 (bold). Panels A and B have matched colours and letters.

We considered all the SNPs associated with DTC on the PubMed database using the following keywords alone and/or in different combinations: papillary thyroid cancer, thyroid cancer, thyroid tumour, DTC, papillary thyroid cancer (PTC), GWAS and association. A total of 171 SNPs were initially selected from 156 studies, including both candidate gene studies and GWAS, demonstrating an association with DTC (*P* < 0.05) (Supplementary Table 1). These SNPs were evaluated for their association with DTC risk in dataset 1 (Supplementary text and Supplementary Table 2). A subset of 34 selected SNPs successfully passed the test and they were genotyped in dataset 2. Fifteen SNPs were considered positive (Supplementary Table 3) and genotyped in dataset 3. These SNPs were used for the Bayesian analysis of population genetic structure and assessment of genetics disease risk using ML algorithms. The selection criteria are shown (Fig. 2) and further explained in the ‘Statistical analysis’ section.

### Statistical analysis

Each genotype was evaluated by the chi-square test for the Hardy–Weinberg equilibrium (HWE) in controls, employing the Bonferroni’s correction (*P* threshold = 1.47 × 10^−3^). The association between the health state and genotypes was evaluated with multivariate logistic regression analysis (MLRA). The model returns the odds ratio adjusted (OR_adj_) for covariates (e.g. sex and age) and their 95% CI with a statistical *P* value of the association. The most likely mode of inheritance was evaluated by performing an extended maximum of the optimal (MAX) tests (30) based on multiplicity-adjusted *P* values for the Cochran–Armitage trend test of the dominant, additive and recessive models.

In order to select SNPs robustly associated with the DTC risk, among the 171 candidates, we carried out a two-stage case/control association study. The first step was performed by evaluating the extent of association of the candidate SNPs with DTC risk obtained in dataset 1 (12). For each SNP, the additive, recessive and dominant models of inheritance were evaluated and SNPs showing a statistically significant association (*P* < 0.05) were passed to the second step, performed on dataset 2.

The selected SNPs served for PRS and weighted PRS (wPRS) calculation, in the three merged datasets. The PRS was built by summing the total number of risk alleles for each subject (attributing the value of 1 to each risk allele). The wPRS was built by assigning to each genotype the relative OR obtained in the GWAS. Then, the ORs were multiplied. For PRS, we assessed the cumulative effect of the independent significant SNPs with an additive model. For each SNP, the genotypes were coded as 0, 1 or 2, indicating the number of risk alleles in the genotype. Then, individuals were grouped according to the total number of risk alleles into quintiles with the lowest group used as the reference. For wPRS, as previously reported (31), the number of risk alleles for each genotype was multiplied for its relative weight, based on the association of the allele with the health state, as: PRS = β1 × 1 + β2 × 2 + [⋯] + βk xk +⋯+ βn xn; where βk is the per-allele log OR for the disease associated with SNP k, xk is the allele dosage for SNP k and n is the total number of SNPs included in the PRS.

### Bayesian statistics for population genetics

We tested the capability of the 15 selected SNPs to provide a DTC genetic signature in the merged datasets 1, 2 and 3. The genetic structure of DTC patients and healthy controls was explored according to methods of Bayesian statistics for population genetics implemented in the STRUCTURE 2.3.4 software (27), as previously described (32). The case/control state of individuals was unknown to the software, which inferred genetic structures using only SNP data. Bayesian analysis and software settings are detailed in the supplementary online material (Supplementary text).

### Machine learning-based analysis

In the preliminary phase of ML algorithm selection, different approaches were tested, namely k-Nearest Neighbours, Naïve Bayes (33), Random Forest, Gradient Boosting (34), AdaBoost (35) and Support Vector Machine algorithms, as implemented in the SciKit-Learn (36) library for Python. The AdaBoost classifier (37) was selected as the best overall algorithm (Supplementary text and Supplementary Fig. 1). We used the SciKit-Learn implementation of the AdaBoost classifier, where the base learner is a Decision Tree classifier with a maximum depth of 1, sometimes referred to as ‘decision stump’. The total number of base estimators was tuned in the range 1–100 (with a step of 1) to maximize ROC–AUC on the test set. The classifier was run on three datasets (Table 1): (1) a training set, used for the training of the algorithm, composed of a randomly extracted 80% of the merged dataset 1 + 3; (2) a test set, for an initial performance evaluation and hyperparameter tuning, composed of the remaining 20% of the merged 1 + 3 dataset; (3) a validation set, corresponding to dataset 2 after pruning missing values, which constitutes a third, unseen dataset used for external validation.

**Table 1:** Summary of training, testing and validation ML datasets.

ML dataset	Origin of dataset^a^	No. of Individuals	Cases (%)	Controls (%)
Training	80% Datasets 1 + 3	1086	58.2	41.8
Testing	20% Datasets 1 + 3	272	62.1	37.9
Validation	100% Dataset 2	201	65.7	34.3

^a^ Summary of datasets after removing individuals with missing data in the genotype (% cases; % controls): dataset 1 = 949 (59.5; 40.5); dataset 2 = 201 (65.7; 34.3); dataset 3 = 409 (57.7; 42.3).

## Results

### Population characteristics

The characteristics of subjects enrolled in the study are summarized (Table 2).

**Table 2:** Characteristics of study population.

	Dataset 1		Dataset 2		Dataset 3	
	Cases (*n* = 649)	Controls (*n* = 431)	Cases (*n* = 234)	Controls (*n* = 101)	Cases (*n* = 404)	Controls (*n* = 392)
Females (%)	507 (78%)	320 (74%)	167 (71%)	61 (60%)	287 (71%)	243 (62%)
Age (years)	37.8 ± 0.85	46.8 ± 0.97	49.7 ± 14.0	43.7 ± 11.4	44.8 ± 12.7	43.8 ± 9.6
Weight (kg)	71.0 ± 1.31	70.4 ± 1.37	74.5 ± 15.0	71.3 ± 16.1	79.3 ± 17.9	69.2 ± 14.5
BMI (kg/m^2^)	25.3 ± 0.39	25.2 ± 0.38	26.9 ± 4.9	26.1 ± 5.0	27.5 ± 4.7	23.9 ± 3.7

Values are expressed as number and percentages (%) or average and standard error.

### SNPs associated with DTC

The overall workflow for identification of SNPs associated with the risk of DTC (Fig. 2) is extensively descripted, and results are provided as online supplementary material (Supplementary text). We selected 171 SNPs associated with DTC with a *P* < 0.05 from the online literature database (Supplementary Table 1), and SNPs associated with the risk of DTC in dataset 1 with a *P* < 0.05 were considered positive (Supplementary Table 2), then were genotyped in dataset 2. All SNPs were in HWE in controls. Among these SNPs, four were robustly associated with the risk of DTC (rs965513, rs3758249, rs7048394 and rs944289) as they accomplished the Bonferroni’s threshold of statistical significance in the combined datasets 1 and 2 (Supplementary Table 3). Three SNPs (rs6759952, rs966423 and rs1203952) were considered highly likely DTC risk markers as they were positive in both datasets at the nominal *P* value of 0.05. Eight SNPs (rs10238549, rs7800391, rs1799814, rs7617304, rs4808708, rs10781500, rs1061758 and rs10877887) were considered as possible DTC risk markers as they were statistically significant at the level of 0.05 in the combined datasets. Thus, we finally selected 15 SNPs strongly associated with DTC in datasets 1 and 2 (Table 3). None of them was in LD with each other (r^2^ < 0.8).

**Table 3:** List of the 15 SNPs associated with DTC in datasets 1 and 2.

SNP ID	Genomic location	Gene	Description
rs965513	chr9:97793827	*PTCSC2/FOXE1*	Papillary thyroid carcinoma susceptibility candidate 2/Forkhead box E1
rs3758249	chr9:97851858	*PTCSC2/FOXE1*	Papillary thyroid carcinoma susceptibility candidate 2/Forkhead box E1
rs7048394	chr9:97843151	*PTCSC2/FOXE1*	Papillary thyroid carcinoma susceptibility candidate 2/Forkhead box E1
rs944289	chr14:36180040	*PTCSC3/LINC00609*	Papillary thyroid carcinoma susceptibility candidate 3
rs6759952	chr2:217406996	*DIRC3*	Disrupted in renal carcinoma 3
rs966423	chr2:217445617	*DIRC3*	Disrupted in renal carcinoma 3
rs1203952	chr20:22633494	*FOXA2*	Forkhead box A2
rs10238549	chr7:110540965	*IMMP2L*	Inner mitochondrial membrane peptidase subunit 2
rs7800391	chr7:110568186	*IMMP2L*	Inner mitochondrial membrane peptidase subunit 2
rs1799814	chr15:74720646	*CYP1A1*	Aryl hydrocarbon hydroxylase
rs7617304	chr3:158745312	*RARRES1*	Retinoic acid receptor responder 1
rs4808708	chr19:17890877	*NIS/SLC5A5*	Solute carrier family 5 member 5
rs10781500	chr9:136374886	*CARD9/SNAPC4*	Caspase recruitment domain-containing protein 9
rs1061758	chr9:34652333	*IL11RA*	Interleukin 11 receptor subunit alpha
rs10877887	chr12:62603400	*LINC01465/MIRLET7I*	Long intergenic non-protein coding RNA 1465/microRNA Let-7i

These 15 SNPs were then genotyped in dataset 3, while the remaining 156 SNPs were considered not associated with the risk of DTCs in our study populations.

### Calculation of polygenic risk scores

The 15 positive SNPs were used for the calculation of PRS and wPRS in the merged data of all three datasets. Subjects were divided in quintiles based on the number of risk alleles and the lowest quintile was used as the reference. The risk increased progressively with the increasing number of risk alleles, up to the value of OR_adj_ = 6.87 (95% CI = 4.9–9.64) for the fifth quintile in the wPRS. All the differences were highly statistically significant both in the PRS and the wPRS, already from the second and third quintile (Table 4 and Supplementary Fig. 2).

**Table 4:** Odds ratio estimates for the 15 SNPs PRS quintiles. DTC state obtained in the three merged datasets was considered, using the bottom quintile (0–20%) as the reference group. The multivariate logistic regression model included the adjustment of ORs for age, BMI and gender. wPRS, weighted polygenic risk score; PRS, unweighted polygenic risk score.

Quintile	wPRS			PRS		
	OR_adj_	95% CI	*P*	OR_adj_	95% CI	*P*
I	Reference			Reference		
II	2.12	1.55–2.91	2.92 × 10−6	1.43	1.04–1.97	0.0282
III	2.52	1.84–3.44	7.02 × 10−9	2.55	1.90–3.40	2.87 × 10−10
IV	3.15	2.30–4.32	9.65 × 10−13	3.04	2.26–4.09	2.02 × 10−13
V	6.87	4.90–9.64	6.12 × 10−29	5.84	4.18–8.15	3.75 × 10−25

### Exploration of individual’s genetic structures according to DTC-related SNPs

**Figure 3:**
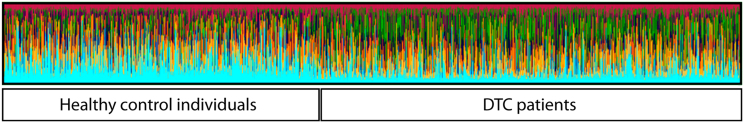
DTC-associated genetic structure of cases and healthy controls. The bar plot was calculated by the STRUCTURE software in the merged datasets 1, 2 and 3. Each individual is represented by a vertical line, in which colours indicate the contribution of each of the k = 5 components to the individual genetic background. Cases and controls were ordered for graphical reasons, showing different genetic profiles at a glance, although indicating a certain degree of admixture.

The association between the risk of DTC and individual genetic profile was explored by the application of Bayesian inference. The 15 SNPs used for the PRS were also employed as an input for the STRUCTURE software, run on the merged datasets 1, 2 and 3. STRUCTURE returned the relative weight of each component in the genetic background of each subject shown as a bar plot (Fig. 3). Five (k = 5) possible genetic structures (components) were found as the most representative of the datasets (26) (Supplementary text). The pattern, calculated using 15 SNPs, reflects at a glance the different DTC-related genetic profile between cases (DTC) and controls.

Output data representing the DTC-related genetic background were analysed to evaluate the quality of Bayesian inference by multiple regression analysis. We identified two components strongly associated with the case/control state: the component 2 (k2) had an F-ratio of 327.66 and the component 3 (k3) had an F-ratio of 106.26 (both *P* < 10^−6^). Results were confirmed by MLRA using the two components as continuous variables. In this case, we found that they were strongly associated with DTC risk, with ORs of 143.4 (95% CI = 52.7–390.2) and 12.2 (95% CI = 5.72–26.1).

### ML-based DTC description using SNP information

**Figure 4:**
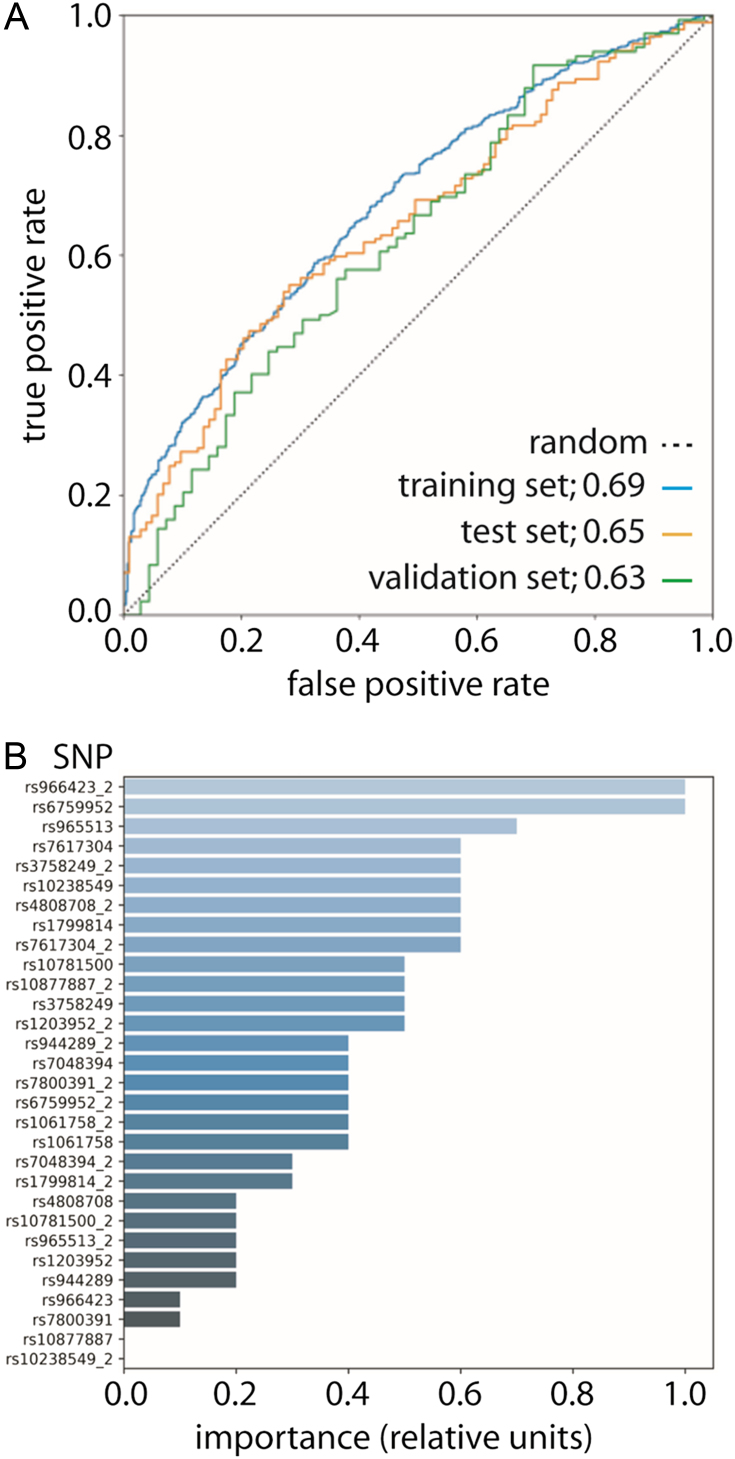
Results from the ML-based DTC prediction and SNP relative importance. (A) ROC curves obtained on all datasets with the AdaBoost model. Dashed line represents random choice. (B) Relative feature importance of all variables (SNPs) in the AdaBoost model. Data normalized to most important feature. Suffix ‘_2’ indicates the second allele. Feature importance is calculated as an average over the individual classifiers used for probability calibration.

The AdaBoost algorithm was found to be the most effective and well-calibrated in classifying individuals (Supplementary text and Supplementary Fig. 3). The classifier was further tuned in terms of the number of base estimators hyperparameter, in a range of 1–100. We found 25 to be an optimal number of base estimators, providing an optimal balance between computational cost and model accuracy (Supplementary text and Supplementary Figs 4 and 5). Additionally, predicted probability calibration was implemented using Platt’s method (38). The detailed metrics of the AdaBoost classifier are reported (Table 5), as well as ROC curves and AUC of all datasets (Fig. 4A).

**Table 5:** Classification metrics of AdaBoost classifier on all datasets.

Metric	Training set	Test set	Validation set
NPV	0.65	0.56	0.52
PPV	0.64	0.66	0.70
Sensitivity	0.88	0.87	0.85
Specificity	0.29	0.27	0.32
Accuracy	64%	64%	67%
F1-score	0.74	0.75	0.77
F0.5-score	0.67	0.70	0.73
F2-score	0.82	0.82	0.82

F_β_ scores are defined as: 
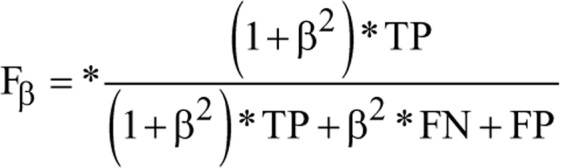
.

NPV, negative predictive value = TN/(TN+FN); PPV, positive predictive value = TP/(TP+FP).

Results clearly highlight that there is no significant overfitting on the training set (Fig. 4A and Table 5), given the reduced differences between the training and test set performance in terms of AUC (0.04), accuracy (0.6%), sensitivity (0.01) and specificity (0.02). This is also confirmed by the 10-fold cross-validation on the train/test splits, which resulted in an average ROC AUC of 0.65 ± 0.03 (s.d.). In addition, when classifying the samples from the external validation set, which again showed comparable classification performance, the model’s ability to generalize on unseen data noticeably emerges. Analysis of the predicted probabilities revealed that they fairly match the real distribution of DTC risk both in the test and in the validation sets (Supplementary text and Supplementary Fig. 5). The performance of other classifiers was weaker than that of the AdaBoost algorithm (Supplementary Figs 3, 6 and 7).

Finally, the importance of each individual SNP allele in the classification by the trained AdaBoost model was evaluated with the aim of exploring the weight of each individual SNP in the identification of the DTC state (Fig. 4B). The most important SNPs, with a relative feature importance greater than 0.6, were rs966423, rs6759952, rs966513, rs7617304, rs3758249, rs10238549, rs4808708 and rs1799814. Interestingly, the top two SNPs, namely rs966423 and rs6759952, were both considered highly likely to be predictive in the SNP selection phase (see paragraph ‘SNPs associated with DTC’).

It is worth mentioning how some of the SNPs which were deemed ‘robustly associated with the risk of DTC’ or ‘highly likely DTC risk markers’ in the association analysis, such as rs7048394, rs944289 and rs1203952, respectively, are not among the top-ranking in terms of importance in the ML model. This is due to the fact that the prediction of the AdaBoost model is based on the given constellation of all the selected SNPs rather than on the SNPs taken individually. Thus, in this specific classification task, the combination of the top-ranking SNPs in Fig. 4 might contain enough information, so that some of the SNPs which were strongly associated to DTC risk in the initial association analysis become progressively less important, or even redundant, and do not improve the overall predictive performance further.

## Discussion

The present study outlined the potential of a minimal selection of 15 DTC-associated SNPs to confirm the disease predisposition. We re-evaluated the candidate risk ‘loci’ described in the literature as individually associated with DTC. Candidates were verified for their association by consulting the results obtained in a previous GWAS (12). The most strongly associated SNPs were *ad hoc* genotyped in 2 independent datasets, accounting for a total of 1131 individuals. The 15 best-performing SNPs were used for the calculation of PRS and wPRS and employed for reconstructing the genetic structure of individuals. Most importantly, we used these SNPs to describe the DTC and healthy control state of individuals using ML. Interestingly, the classification using the AdaBoost algorithm showed fair performance in the test set, with accuracies as high as 64%, as well as in the external validation set, settling at 67% (Table 5). Considering that the ROC AUC is a performance measurement for the classification (39), we may assume to have detected a minimal pool of SNPs consistently contributing to the risk of developing DTC in our Italian dataset, as an example of polygenic disease. This has been further confirmed by the fair confidence of the AdaBoost in classifying the disease state, as highlighted by the positive predictive value of up to 0.7 on the external validation set. Our results provide a substantial improvement in understanding the impact of genetics on DTC, which until now could be estimated by PRS and could explain only 11% of the total genetic variability linked to the disease (24, 23). Moreover, the 15 selected SNPs describe the inner genetic structure of sampled individuals when assessed using Bayesian inference from population genetics. We evaluated whether this method would decipher differences between cases and controls, assigning a phenotype-specific genetic footprint to individuals, with a quantitative approach. Individuals were distributed among two subpopulations with different genetic patterns, following the DTC or healthy control state, although a certain degree of admixture was found. This analysis confirmed that the selected SNPs are representative of the genetic signature linked to the disease. When using these SNPs for an ML-based analysis of DTC, we obtained a fair classification power.

Overall, we found six SNPs within the *FOXE1,PTCSC3-LINC00609, FOXA2* and *DIRC3* genes robustly associated with the risk of DTC or categorized as highly likely risk factors, confirming they are well-established predisposing factors for DTC (8, 11, 12, 13, 14, 40, 41). SNPs in the *DIRC3* (rs966423, rs6759952) and *FOXE1* (rs3758249) genes were also highly relevant features for DTC risk. Other eight SNPs, such as those falling within the *CYP1A1, NIS*-*SLC5A5, IL11RA* and *let-7i/LINC01465* genes (42, 43, 44, 45, 46, 47, 48, 49, 50, 51, 52, 53, 54, 55, 56, 57, 58, 59, 60), did not replicate formally in the second stage of the study, although they maintained or reinforced the statistical significance of the GWAS in the combined analysis. One SNP falling within the *CYP1A1* gene (rs1799814) was also highly relevant for DTC predisposition. Interestingly, there is relative lack of knowledge on the role of the remaining three genes, i.e. *IMMP2L*, *RARRES1* and *CARD9-SNAPC4* in thyroid cancer. However, since the selected SNPs were associated with DTC in the combined analysis, they could be reasonably involved in the aetiology of the disease. They have been previously involved in the regulation of cell metabolism, oestrogen physiology, tumour suppression or progression and autoimmune diseases (61, 62, 63, 64, 65, 66). These three genes are certainly interesting for future studies in connection with DTC in the future. The remaining 137 SNPs were not confirmed in dataset 1, while the other 19 SNPs positive in the GWAS were not confirmed in dataset 2. They could have been detected as the consequence of chance findings in underpowered studies published in the literature, resulting as false or weakly positive signals. An extensive discussion of these gene SNPs is provided as supplementary material (Supplementary text).

Considering the 15 most associated SNPs, we also calculated PRS and wPRS, confirming that the disease risk increases together with the number of risk alleles. An OR of 6.9 (95% CI = 5.4–8.8) for the top 10th decile was found based on a 10-SNPs model, including SNPs falling within *PCNX2*, *DIRC3*, *LRRC34*, *EPB41L4A*, *NRG1*, *PTCSC2*, *STN1*-*SLK*, *PTCSC3 LINC00609*, *MBIP* and *SMAD3* genes. Our results are in agreement with previous studies concluding that the genetic predisposition to PTC may be resumed by only 10 SNPs, found by wPRS analysis and accounting for between 8 and 11% of the total variability (24, 23). In particular, that study had only three markers in LD with SNPs found herein and lacking the requisites for being selected and run on ML analysis (Supplementary text). It is correct to specify that previous studies only studied PTC and not DTC. However, we believe the comparison is feasible as PTC accounts for at least 85% of all thyroid cancers. Similar OR values were found in a previous GWAS performed using a 11-SNPs signature (13), which shared only the *DIRC3* gene region with our proposed signature. Taken together, these data indicate that unknown, low-penetrance SNPs contributing to genetic predisposition to DTC may be discovered using different approaches. A recent study on the Korean population found lower ORs (1.46 and 1.56 for unweighted PRS and ywPRS, respectively), but considering only six SNPs associated with thyroid cancer (67).

Obviously, our study is limited by the fact of not having considered all the possible SNPs associated with DTC in the literature but only those associated with the DTC risk in the GWAS enrolling subjects of dataset 1. Therefore, classification algorithms relied on the genetic information alone. Data about the exposure to important risk factors, such as ionizing radiation and family history of DTC, were only available for a subset of the study population (not shown). Therefore, they could not be considered in the statistical analyses and for the construction of ML models. Another issue may consist in the ethnicity of datasets used herein, which consists of Italian individuals. The association between these SNPs and the disease would be explored in individuals of different ethnicity. Finally, in case/control studies, proper sample selection is crucial to attain robust disease prediction: individuals recorded as healthy controls might develop DTC even in older age, even if subjects had no thyroid abnormalities at the time of ultrasound analysis. Such individuals should be considered as ‘spurious negatives’. Similarly, young DTC individuals might have seen the development of the disease following causes beyond the genetic predisposition, such as exposure to ionizing radiation and might thus represent ‘spurious positives’. These aspects represent confounding factors when attempting to extrapolate the genetic footprint of the disease used to build ML models. For the reasons listed earlier, the direct clinical impact of our result is limited. It has yet to be clarified which other genetic markers cover the remaining slice of heritability or predisposition. Then, it is necessary to analyse genetics together with other environmental risk factors, some of which are difficult to measure, such as exposure to radiation or pollutants.

In conclusion, we described a procedure based on a combined PRS and ML approach that allows a fair description of the case or control state based solely on the individual genetic background. This analysis provided evidence for a new, restricted selection of 15 SNPs associated with the risk of DTC, extending the series previously found using different approaches (24) and further delineating the genetic signature of the disease in our Italian dataset. Further developments might aim to implement and refine the reported methodology with more covariates and might improve the overall accuracy.

## Supplementary Material

Supplementary Material

Supplementary table S1. List of SNPs associated to DTC in different world populations (p<0.05).

Supplementary table 2. List of 171 SNPs selected from literature and evaluated for their statistical association with the risk of DTC in the dataset 1. Querid SNPs g= directly genotyped; i= imputed from 1000Genomes database (www.internationalgenome.org); Best P-value (model) a=additive, when the model tests whether heterozygotes have intermediate risk between common and rare homozygotes; d=dominant, when heterozygotes are lumped together with rare homozygotes and these are compared versus common homozygotes; r= recessive, when rare homozygotes are compared versus heterozygotes lumped together with common homozygotes.

Supplementary Table 3. Association analyses for 34 SNPs genotyped in the dataset 2 set alone (r) and combined (c) with the dataset 1. SNPs are listed as reported in table 1. Odd Ratios (adjusted for sex, age, smoking habit, body mass index) are provided with their 95% confidence intervals (ORadj; 95% CI). Associations are underlined when statistically significant at nominal level of 0.05 and evaluated for the Bonferroni’s correction (p-threshold=1.47x10exp-3).

Supplementary table S4. Classification metrics of Gaussian Naïve Bayes classifier on all datasets.

Supplementary Table S5: method of calculation of unweighted and weighted polygenic risk score (PRS). The unweighted PRS was built by summing the total number of risk alleles for each subject (attributing the value of 1 to each risk allele). In the weighted PRS, all the SNPs contribute to the total PRS according to their association with the risk. It was built by assigning to each genotype the relative OR obtained in the GWAS. Then the ORs were multiplied.

## Declaration of interest

The authors declare that there is no conflict of interest that could be perceived as prejudicing the impartiality of the research reported.

## Funding

K H was supported by the Horizon 2020 Program of the European Union (grant 856620).

## Author contribution statement

G B participated to study design, data collection, analysis and interpretation, manuscript writing and revision. C L, E P, F N and M Sit were involved in data collection. F T revised the manuscript and was involved in data interpretation and intellectual content. G M, R S and F G was involved in data analysis. A F, K H and R E participated to data collection, interpretation of results and revised the intellectual content of the manuscript. Cristina Romei was involved in data collection and analysis. E A Z and M A D participated to study design, data analysis and interpretation, manuscript writing and revision. M S was involved in interpretation of results and revised the intellectual content of the manuscript. S L and L C conceived the study, participated to study design, data collection, analysis, interpretation and intellectual content, manuscript writing and revision.
