# Supplementary material for: Genetic signature of differentiated thyroid carcinoma susceptibility: a machine learning approach
Source: Eur Thyroid J. 2022 Aug 17;11(5):e220058. doi: 10.1530/ETJ-22-0058 (PMC9513665; doi:10.1530/ETJ-22-0058)
Supplement: Supplementary Material [file supplementary_material.pdf]

# SUPPLEMENTARY TEXT AND FIGURES

## SUPPLEMENTARY METHODS

### DNA extraction

Genomic DNA was extracted from blood samples by the automated extractor EZ1 Advanced XL using the EZ1 DNA Blood Kit (Qiagen, Hilden, Germany). DNA concentrations and purity were determined by the NanoDrop™ 2000 spectrophotometer (Thermo Fisher Scientific, Waltham, MA, USA). Samples from datasets 2 and 3 were shipped in dry ice for SNP genotyping, which was performed by iPLEX® assay (Life & Brain GmbH, Bonn, Germany) as an on-demand service including primer probes design and validation.

### SNP selection for association in the dataset 1

The list of 171 SNPs selected from literature was evaluated for their statistical association with the risk of DTC in the dataset 1 (Supplementary table S1). In Supplementary Table S1 significant SNPs are ranked in ascending order of the P-values of the association tests referring to the best model of inheritance (r=recessive; d=dominant; a=additive). The non-significant SNPs are reported in alphabetical order of the gene names. SNPs within the same block of linkage *disequilibrium* (LD) are reported consecutively. Within each gene, the blocks of LD are numbered progressively. For each SNP, the parameter $r^2$ is showed as measure of LD and it is referred to the pair-wise distance to the first listed SNP within the block. SNPs associated at nominal level of <0.05 were further genotyped in a replication set (second stage, last column), with the exception of rs1048943 because the low minor allele frequency (MAF=0.021) could not provide the adequate statistical power.

### Bayesian statistics for population genetics

In the “assisted” analysis, the STRUCTURE software runs using genetic data of 15 SNPs found to be associated with DTC after the selection procedure. The case/control *status* of all individuals was

known to the software and used to assist inference using the LOCPRIOR model. The Bayesian inference was launched using Markov chain Monte Carlo (MCMC) simulations after burn-in periods. The number of possible subpopulations (K) ranged from 1 to 10, for a total of 20 iterations. The most probable K number was calculated using the online tool STRUCTURE Harvester [1] applying the ΔK method [2]. Results from the 20 iterations were packaged by the Cluster Markov Packager Across K (CLUMPAK) online tool [3] using the default settings. Separate runs on unmerged datasets were also performed.

To test if this method was suitable for diverse datasets, we repeated the same protocol using dataset 3 (Table 1) as input, consisting of a balanced pool of 796 individuals, and report results using the same metrics.

**Machine Learning-based disease analysis**

The performance of a Machine Learning (ML) model in describing the patient/control state was evaluated. To evaluate the performance of different ML methods, an extended dataset was built by merging the two largest datasets (1 and 3), to obtain a highly informative and balanced pool of training data: randomly chosen 80% of this extended set was used to train ML models, with the case or control state known to the classifier, and the remaining 20% of the subjects was used for an initial performance evaluation, with the original labels hidden. After finding the most effective ML algorithms for the present scenario, i.e using the 15 SNPs as input variables, their predictive capability was validated on the dataset 2, to quantitatively assess their generalization capabilities. The whole procedure is summarized in the main article (Figure 1).

ML methodologies were applied to predict the patients' disease state based solely on the information coming from the previously selected 15 SNPs. Thus, we used the three datasets (Figure 1A) as inputs for different types of classifiers. To prepare the data and increase ML performance and robustness, any individual with missing data in the genotype was pruned from all datasets, to avoid that ML algorithms interpret missing data as a genetic information. SNP variables were

encoded as follows: firstly, each SNP genotype was split into its two alleles, thus yielding a total of 30 variables, for which ordinal variable encoding was used, to convert the four-letter genetic code (A-T-G-C) to numerical values (1-2-3-4). Since individuals with missing values were pruned from the datasets, no other numerical value was present in the training data. The final composition of the three datasets after the pruning of missing values is summarised (Table 1).

For the subsequent ML implementation, three datasets were extracted: (1) a training set, used for the training of the algorithm, composed of a randomly extracted 80% of the merged dataset 1+3; (2) a test set, for an initial performance evaluation and hyperparameter tuning, composed of the remaining 20% of the merged 1+3 dataset; (3) a validation set, corresponding to dataset 2 after pruning missing values, which constitutes a third, unseen dataset used for external validation. Indeed, it should be noted that the earlier discovery of potentially informative SNPs to genotype (see paragraph "SNP selection") did not involve any ML algorithm: since the selection was not performed based on ML performance outcome, the validation set has never been fed to the algorithm at any earlier stage.

Conversely, the rationale behind merging datasets 1 and 3 to obtain a large training set lies in the uniformity of sampling location and the goal to obtain a large pool of data for improved ML training. A graphical summary of the ML pipeline is reported (Supplementary figure S1).

The performance of the tested ML algorithms was evaluated both on the internal test set and on the external validation set, to ensure the generalization ability of the trained algorithms and to assess the tendency towards overfitting. More in detail, the following metrics were calculated and reported: Receiver Operating Characteristic area under the curve (ROC-AUC), Accuracy, Positive Predictive Value (PPV), Negative Predictive Value (NPV), Sensitivity, Specificity, F1-Score, F0.5-score, F2-score.

In the preliminary phase of ML algorithm selection, different approaches were tested, namely k-Nearest Neighbors (kNN), Naïve Bayes (NB) [4], Random Forest (RF), Gradient Boosting (GB)

[5], AdaBoost (AB) [6] and Support Vector Machine (SVM) algorithms, as implemented in the SciKit-Learn [7] library for Python. The best methodology was initially chosen based on the ROC AUC value, and subsequently further optimised for optimal performance in a hyperparameter tuning stage (Supplementary figure S1). A ROC curve describes the relationship between the sensitivity and specificity of a test by plotting the two against one another while varying the evaluation threshold, which determines the outcome of a test. Sensitivity and specificity are inversely related: as one increases the other decreases. Conventionally, since both values range between 0 and 1, the sensitivity (true positive rate) is plotted against 1 minus the specificity (false positive rate). The plot is, therefore, in essence, a representation of the trade-off between detecting true and false positive cases.

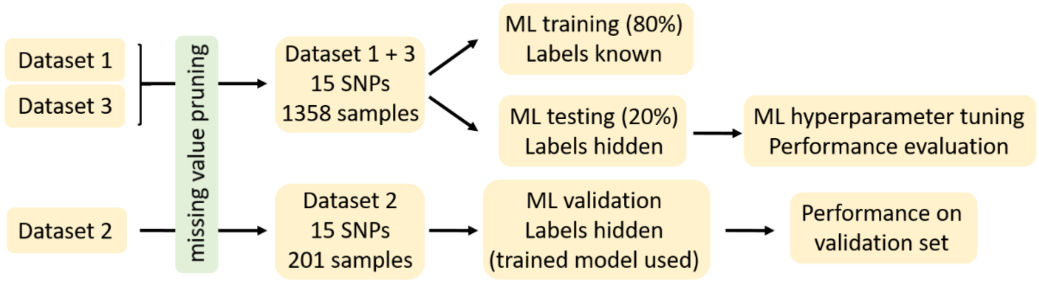


Supplementary figure S1. Pipeline of ML data analysis. Randomly chosen 80% of the merged datasets 1 and 3 served for training the ML algorithm, while the remaining, 20% was the test set. The dataset 2 was used as an external validation set. Individuals with missing SNP data were excluded from the ML analysis.

Supervised ML approaches was applied here to classify the case/control state of the individuals. Three datasets were used for the training, testing and external validation of ML algorithms, constituted respectively by 80% of merged datasets 1+3, 20% of merged datasets 1+3, and dataset 2. Among the tested algorithms, namely kNN, NB, RF, GB, AB and SVM, the most suitable was chosen based on the ROC-AUC, Sensitivity, Specificity, Accuracy and prediction probability calibration on both the test and validation sets (Table 5).

After analysing the performances of the different classifiers (Supplementary Figure S1 and S3), the Naïve Bayes classifier and the Adaptive Boosting (AdaBoost) classifier were chosen for further performance tuning and evaluation.

The Naïve Bayes classifier is a supervised learning algorithm relying on Bayes' theorem and assuming naïve conditional independence between features. In detail, in this study a Gaussian Naïve Bayes algorithm was used, as implemented in the SciKit-Learn library for Python.

The AdaBoost algorithm was implemented using decision stumps, i.e. decision trees with depth of 1, as base estimators, and the number of the latter was tuned to obtain the best AUC while at the same time optimising the computational effort. The performance of both classifiers was first evaluated on the test set, and subsequently on dataset 3 used as an external validation set, to further check the performance of classification of unseen data. Furthermore, the individual metrics including PPV, NPV, specificity, sensitivity and accuracy were analysed along with the prediction probability calibration to choose the final model, reported in the main text. Finally, the AdaBoost algorithm was found to be the most effective and well-calibrated in classifying individuals (Supplementary figure S3).

## SUPPLEMENTARY RESULTS

### SNPs associated with DTC

Considering that the candidate SNPs were associated with the risk of DTC in previous studies and that, in the present work, a further validation step was carried out, we considered positive the SNPs associated with the risk of DTC with a P-value below the classical statistical significance level of 0.05. Thus, 34 candidate SNPs were associated with the risk of DTC in the dataset 1 and were further evaluated in the dataset 2. The results of genotyping of this series were evaluated alone and combining the populations. Some of the SNPs were clearly associated with the risk of DTC in the dataset 2, also accomplishing the more stringent statistical significance threshold following Bonferroni's correction. Other SNPs were less clearly associated with the risk. For example, some

were associated in both populations at the level of 0.05 (e.g. rs6759952), others were associated only when the two populations were combined (e.g. rs10238549). We were aware that setting a stringent threshold could lead to discharge truly positive SNPs. Thus, we relaxed the selection, by applying the criteria reported in the flowchart (Figure 2) and we drew the following conclusions:

(a) 4 SNPs (rs965513, rs3758249, rs7048394, rs944289) were robustly associated with the risk of DTC, as they accomplished the Bonferroni's threshold of the statistical significance in dataset 2;

(b) 3 SNPs (rs6759952, rs966423, and rs1203952) were considered highly likely markers of risk of DTC as they were positive in both datasets at the nominal P-value of 0.05;

(c) 8 SNPs (rs10238549, rs7800391, rs1799814, rs7617304, rs4808708, rs10781500, rs1061758, and rs10877887) were considered as possible marker risk of DTC, as they were statistically significant at the level of 0.05 in the GWAS and in the merged dataset;

(d) the remaining 156 SNPs were considered not associated (at least in our Italian populations) with the risk of DTC: 137 were negative in the GWAS, 19 were classed as "likely negative" according to the results of the dataset 2.

Considering the number of risk alleles, and weighing each allele based on the measured OR, the ROC curve analysis demonstrated an AUC of 0.65, with 10 alleles as the cut-off to better predict the risk of having DTC.

**Polygenic risk score: OR calculation**

OR was calculated in each quintile under both additive and weighted models, in the three merged datasets (Table 4; Supplementary figure S2).

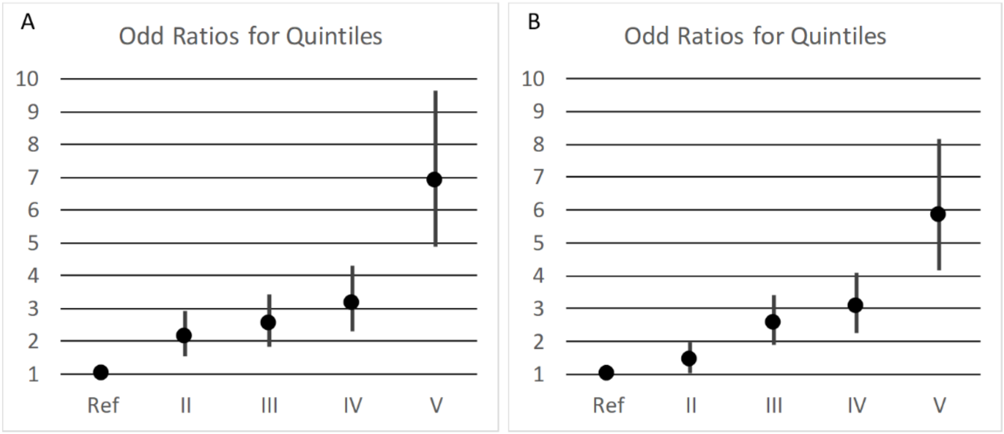


Supplementary figure S2. ORs calculated for each quintile. A) Multiplicative model. B) Additive model. The quintile number is indicated in the x-axis. Dots are adjusted ORs, while bars are the 95% CIs.

## Choice of ML model

The ROC curve of different types of classifiers (kNN, NB, RF, GB, AB, SVM) on both the test and external validation sets are reported (Supplementary figure S3).

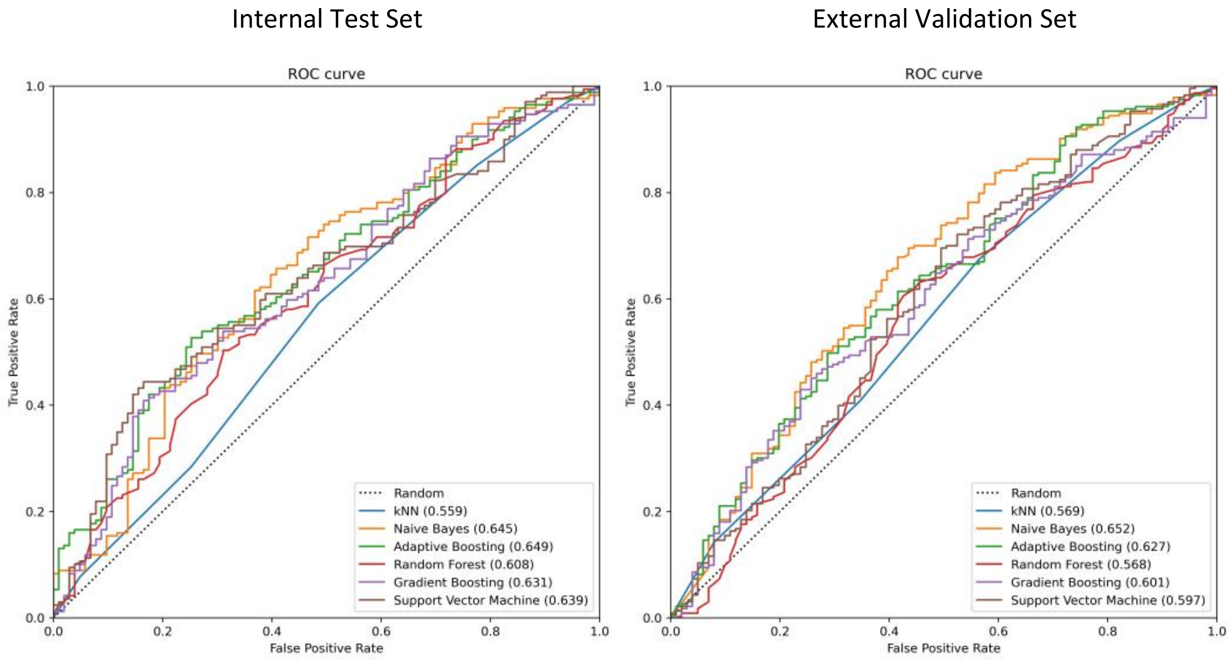


Supplementary figure S3: ROC curves of different types of classifiers. Inset reports the legend and AUC values in brackets.

Based on the initial AUC value, the AdaBoost classifier was chosen for further performance assessments and optimization. For the AdaBoost classifier, we tuned the hyperparameter regarding the number of base estimators, in a range from 1 to 100 (Supplementary figure S4).

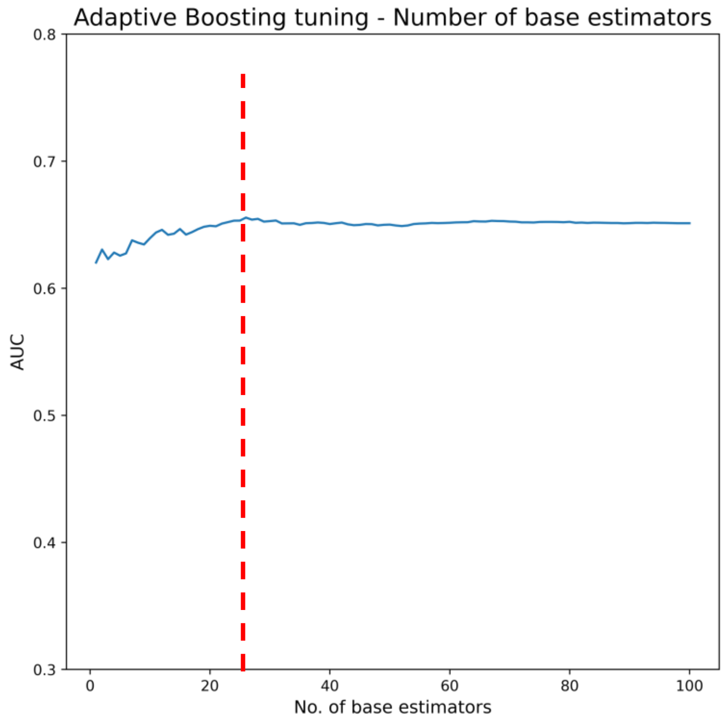


Supplementary figure S4. Tuning of number of base estimators of AdaBoost classifier based on ROC-AUC maximization. Red dashed line represents optimal value of 25.

As shown (Supplementary figure S4), we found 25 base estimators to be an optimal compromise between ROC-AUC maximization and computational cost. Further details and results regarding the optimized AdaBoost classifier are reported in the main text.

## Calibration of the AdaBoost classifier

A drawback of the classifier appears related to the weak identification of the negative class (i.e. controls), as emerges from the comparably low specificity on all datasets, which has a detrimental effect also on the ROC AUC, on the negative predictive value and on overall accuracy, which however also depends on the underlying class balance of the dataset being classified. Conversely,

when factoring in the class distribution (DTC vs. controls) of the underlying datasets being classified, the algorithm shows a fair confidence in predicting the DTC *status* (PPV = 0.7 on the external validation), at the cost of a comparably poorer specificity. By using Platt scaling on the predicted probabilities, the distribution of the latter fairly matches the real distribution of DTC risk both in the test set and in the external validation set (Supplementary figure S5). This was not the case for the Naïve Bayes classifier.

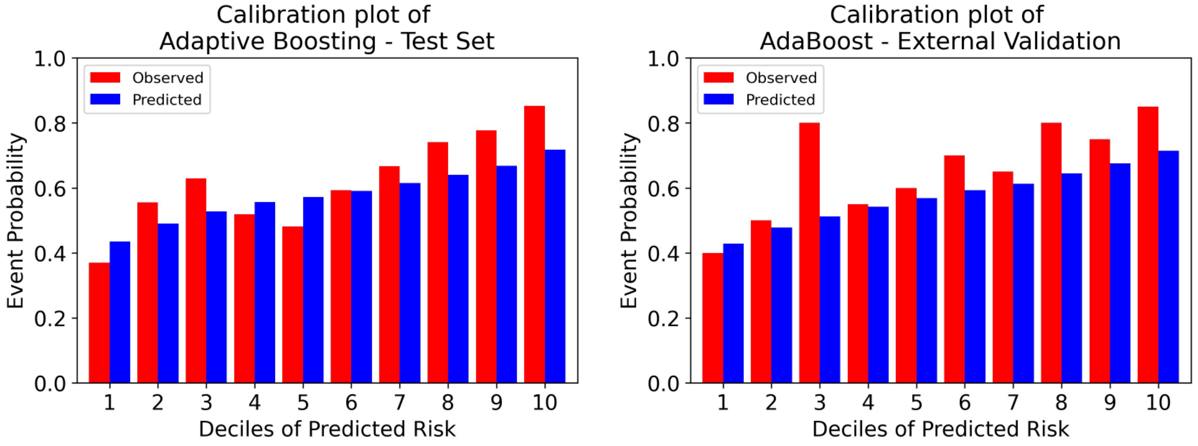


Supplementary figure S5. Calibration plot of the AdaBoost classifier on the test set (left) and the external validation set (right). Blue bars represent the mean predicted probability of DTC in each risk decile. Red bars represent the actual probability of observing DTC in each decile.

## Naïve Bayes classifier

The Naïve Bayes classifier represents the second tested ML model to predict the case/control *status* using only SNP data, relying on Bayesian statistics. After training the Gaussian Naïve Bayes classifier on the dataset described in the Methods section, we deployed the classifier on the test set and obtained an AUC value of 0.65, with an overall classification accuracy of 57% and an F1 score of 0.57 (Supplementary table S4). The AUC of the precision-recall curve was 0.74, and a further 10-fold cross-validation yielded an average ROC AUC of 0.64 ± 0.02 (SD).

We again assessed the ability of the model to generalize to a further dataset containing unseen data by deploying the trained NB classifier on dataset 2 (Supplementary figure S6). Here, an AUC value of 0.67 was obtained, with an overall accuracy of 56% and an F1 score of 0.56.

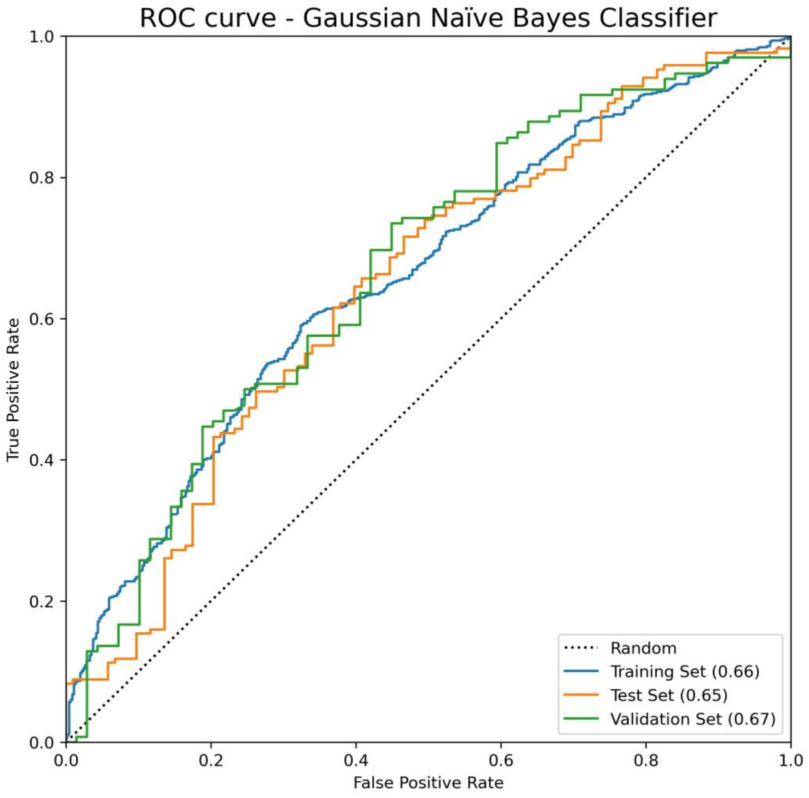


Supplementary figure S6. ROC curves obtained on all datasets. Dashed line represents random choice. A 10-fold cross-validation ROC AUC of 0.65 ± 0.03 (SD) was found.

Again, metrics from the classification of the test and validation datasets highlight no significant overfitting on the training set, with the AUC on the validation being slightly higher than the AUC obtained on both the training and test sets. In contrast to what was found for the AdaBoost classifier, this classifier appears to have a tendency towards classifying individuals into the negative class (i.e. controls), as highlighted by the higher specificity (0.76 and 0.81 on the test and validation sets, respectively). However, when factoring in the prevalence of each class in the datasets – i.e. when considering NPV and PPV values instead of specificity and sensitivity – the confidence in the positive prediction is higher (PPV is 0.76 on the test set, 0.81 on the validation set) if compared to

the negative prediction (NPV values are 0.46 and 0.43 on the test and validation datasets respectively) (Supplementary figure S7).

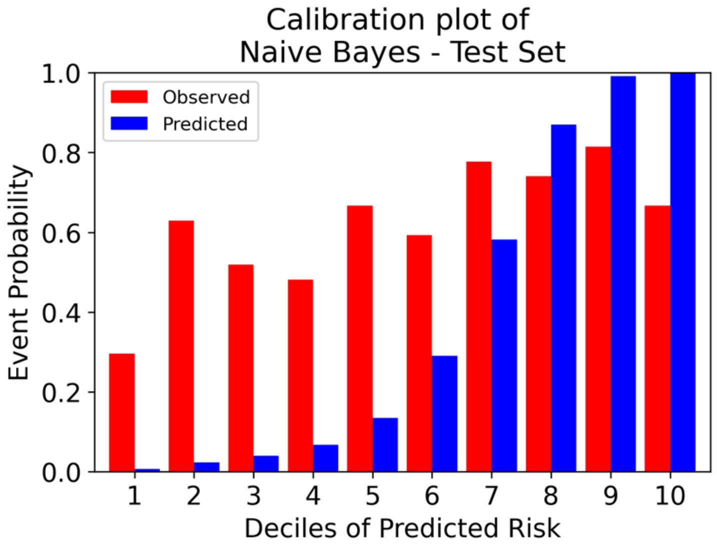


Supplementary Figure S7. Calibration plot of the trained Naïve Bayes classifier on the test set. Blue bars represent the mean predicted probability of DTC in each risk decile. Red bars represent the actual probability of observing DTC in each decile (ground truth).

While the Naïve Bayes classifier showed comparable performance to the AdaBoost classifier in terms of raw AUC values, the calibration of the predicted probability turned out comparably poor (Supplementary figure S6). Specifically, this translates into a classifier which is overconfident in predicting the negative class, as highlighted by the skewed predicted probability towards higher deciles (Supplementary figure S7) and by the comparably high specificity and low NPV. These considerations justify the eventual choice of the AdaBoost algorithm in the light of its better probability calibration and thus increased robustness when facing imbalanced classification tasks.

## SUPPLEMENTARY DISCUSSION

Three SNPs within the *FOXE1* gene (rs965513, rs3758249, rs7048394) and one in *PTCSC3-LINC00609* (rs944289) robustly associated with the risk of DTC. Moreover, other two SNPs within

*DIRC3* (rs6759952 and rs966423) and one in *FOXA2* (rs1203952) were categorized as highly likely risk factors for DTC. Indeed, there is plenty of literature about the role of *FOXE1*, *PTCSC3*, *DIRC3*, and *FOXA2* in affecting the individual susceptibility to thyroid cancer [8-14], revealing they are well-established predisposing factors for DTC. Other eight SNPs, falling within seven genes and known as possible risk factors for DTC, did not replicate formally in the second stage of the study. However, they maintained or reinforced the statistical significance of the GWAS in the combined analysis. For 4 of these genes there is convincing evidence from literature for their role in thyroid tumorigenesis: SNPs within *CYP1A1* (rs1799814), *NIS-SLC5A5* (rs4808708), *IL11RA* (rs1061758), and *let-7i/LINC01465* (rs10877887) are highly likely to be true markers of individual predisposition to DTC, as well as to other cancers [15-33].

Interestingly, there is relative lack of knowledge on the role of the remaining three genes, i.e. *IMMP2L* (rs10238549, rs7800391), *RARRES1* (rs7617304), and *CARD9-SNAPC4* (rs10781500) in thyroid cancer. However, since the selected SNPs were associated with DTC in the combined analysis, they could be reasonably involved in the aetiology of the disease. *IMMP2L* encodes for the inner mitochondrial membrane peptidase subunit 2 and it is a key-molecule linking cellular senescence to metabolism and cell death signalling pathways [34]. It could be hypothesized that polymorphic variants within *IMMP2L* might affect thyrocyte ability to undergo senescence, therefore sustaining immortalization. *RARRES1*, also known as *TIG1* (tazarotene-induced gene 1), encodes for a retinoic acid regulated carboxypeptidase inhibitor. According to the function of a tumor suppressor, *RARRES1* is hypermethylated in multiple cancers [35,36] and its expression is modulated by estrogens [37], suggesting a possible role in the known gender difference of DTC incidence. Finally, *CARD9* encodes for the caspase recruitment domain-containing protein 9 and it is a central adaptor protein of innate immune responses to extracellular pathogens via releasing of active cytokines from the immune cells. In fact, suppression of T lymphocyte functioning was found in *CARD9*-knockout mice and was linked to lung cancer progression [38]. A role of *CARD9* in autoimmunity could be also evoked. Since it is involved in autoantibody-induced autoimmune

disease [39], it could be hypothesized that the association with rs10781500 reflects a low-grade chronic state of autoimmunity against thyroid, predisposing carriers of the risk alleles to DTC.

The remaining 137 SNPs were not confirmed in the dataset 1, while other 19 SNPs positive in the GWAS were not confirmed in the dataset 2, at least in Italian individuals. They could have been detected as the consequence of chance findings in previously published underpowered studies, resulting as false or weakly positive signals.

A previous study evaluating PRS found 10 SNPs describing genetic predisposition to DTC for 8-11% of the total variability [40]. Among those SNPs, only three (rs2466076, rs1588635 and rs116909374) are in *linkage disequilibrium* with markers evaluated in our analysis (rs2439302, rs7028661 and rs925489, respectively) but discarded during the selection procedure. The rs1588635 falls within the *PTCSC2* gene, suggesting it as being a hot spot for genetic predisposition to DTC risk, while the others belong to genomic regions not represented by our 15-SNPs signature. Therefore, we may suppose that a number of unknown, low-penetrance SNPs contribute to DTC genetic predisposition and they may be discovered using different approaches and populations.

## REFERENCES


1. Earl DA, vonHoldt BM (2012) STRUCTURE HARVESTER: a website and program for visualizing STRUCTURE output and implementing the Evanno method. Conservation Genetics Resources 4 (2):359-361. doi:10.1007/s12686-011-9548-7

2. Evanno G, Regnaut S, Goudet J (2005) Detecting the number of clusters of individuals using the software STRUCTURE: a simulation study. Molecular ecology 14 (8):2611-2620. doi:10.1111/j.1365-294X.2005.02553.x

3. Kopelman NM, Mayzel J, Jakobsson M, Rosenberg NA, Mayrose I (2015) Clumpak: a program for identifying clustering modes and packaging population structure inferences across K. Molecular ecology resources 15 (5):1179-1191. doi:10.1111/1755-0998.12387

4. Ng AY, Jordan MI (2001) On discriminative vs. generative classifiers: a comparison of logistic regression and naive Bayes. Paper presented at the Proceedings of the 14th International Conference on Neural Information Processing Systems: Natural and Synthetic, Vancouver, British Columbia, Canada,

5. Friedman JH (2001) Greedy function approximation: A gradient boosting machine. The Annals of Statistics 29 (5):1189-1232, 1144

6. Freund Y, Schapire RE A desicion-theoretic generalization of on-line learning and an application to boosting. In, Berlin, Heidelberg, 1995. Computational Learning Theory. Springer Berlin Heidelberg, pp 23-37

7. Pedregosa F, Varoquaux G, Gramfort A, Michel V, Thirion B, Grisel O, Blondel M, Prettenhofer P, Weiss R, Dubourg V, Vanderplas J, Passos A, Cournapeau D, Brucher M, Perrot M, Duchesnay É (2011) Scikit-learn: Machine Learning in Python. J Mach Learn Res 12 (null):2825–2830

8. Gudmundsson J, Sulem P, Gudbjartsson DF, Jonasson JG, Sigurdsson A, Bergthorsson JT, He H, Blondal T, Geller F, Jakobsdottir M, Magnusdottir DN, Matthiasdottir S, Stacey SN, Skarphedinsson OB, Helgadottir H, Li W, Nagy R, Aguillo E, Faure E, Prats E, Saez B, Martinez M, Eyjolfsson GI, Bjornsdottir US, Holm H, Kristjansson K, Frigge ML, Kristvinsson H, Gulcher JR, Jonsson T, Rafnar T, Hjartarsson H, Mayordomo JI, de la Chapelle A, Hrafnkelsson J, Thorsteinsdottir U, Kong A, Stefansson K (2009) Common variants on 9q22.33 and 14q13.3 predispose to thyroid cancer in European populations. Nature genetics 41 (4):460-464. doi:10.1038/ng.339

9. Gudmundsson J, Sulem P, Gudbjartsson DF, Jonasson JG, Masson G, He H, Jonasdottir A, Sigurdsson A, Stacey SN, Johannsdottir H, Helgadottir HT, Li W, Nagy R, Ringel MD, Kloos RT, de Visser MC, Plantinga TS, den Heijer M, Aguillo E, Panadero A, Prats E, Garcia-Castaño A, De Juan A, Rivera F, Walters GB, Bjarnason H, Tryggvadottir L, Eyjolfsson GI, Bjornsdottir US, Holm H, Olafsson I, Kristjansson K, Kristvinsson H, Magnusson OT, Thorleifsson G, Gulcher JR, Kong A, Kiemeney LA, Jonsson T, Hjartarson H, Mayordomo JI, Netea-Maier RT, de la Chapelle A, Hrafnkelsson J, Thorsteinsdottir U, Rafnar T, Stefansson K (2012) Discovery of common variants associated with low TSH levels and thyroid cancer risk. Nature genetics 44 (3):319-322. doi:10.1038/ng.1046

10. Köhler A, Chen B, Gemignani F, Elisei R, Romei C, Figlioli G, Cipollini M, Cristaudo A, Bambi F, Hoffmann P, Herms S, Kalemba M, Kula D, Harris S, Broderick P, Houlston R, Pastor S, Marcos R, Velázquez A, Jarzab B, Hemminki K, Landi S, Försti A (2013) Genome-wide association study on differentiated thyroid cancer. The Journal of clinical endocrinology and metabolism 98 (10):E1674-1681. doi:10.1210/jc.2013-1941
11. Figlioli G, Köhler A, Chen B, Elisei R, Romei C, Cipollini M, Cristaudo A, Bambi F, Paolicchi E, Hoffmann P, Herms S, Kalemba M, Kula D, Pastor S, Marcos R, Velázquez A, Jarząb B, Landi S, Hemminki K, Försti A, Gemignani F (2014) Novel genome-wide association study-based candidate loci for differentiated thyroid cancer risk. The Journal of clinical endocrinology and metabolism 99 (10):E2084-2092. doi:10.1210/jc.2014-1734
12. Son HY, Hwangbo Y, Yoo SK, Im SW, Yang SD, Kwak SJ, Park MS, Kwak SH, Cho SW, Ryu JS, Kim J, Jung YS, Kim TH, Kim SJ, Lee KE, Park DJ, Cho NH, Sung J, Seo JS, Lee EK, Park YJ, Kim JI (2017) Genome-wide association and expression quantitative trait loci studies identify multiple susceptibility loci for thyroid cancer. Nature communications 8:15966. doi:10.1038/ncomms15966
13. Wang YL, Feng SH, Guo SC, Wei WJ, Li DS, Wang Y, Wang X, Wang ZY, Ma YY, Jin L, Ji QH, Wang JC (2013) Confirmation of papillary thyroid cancer susceptibility loci identified by genome-wide association studies of chromosomes 14q13, 9q22, 2q35 and 8p12 in a Chinese population. Journal of medical genetics 50 (10):689-695. doi:10.1136/jmedgenet-2013-101687
14. Kim HS, Kim DH, Kim JY, Jeoung NH, Lee IK, Bong JG, Jung ED (2010) Microarray analysis of papillary thyroid cancers in Korean. The Korean journal of internal medicine 25 (4):399-407. doi:10.3904/kjim.2010.25.4.399
15. Figlioli G, Elisei R, Romei C, Melaiu O, Cipollini M, Bambi F, Chen B, Köhler A, Cristaudo A, Hemminki K, Gemignani F, Försti A, Landi S (2016) A Comprehensive Meta-analysis of Case-Control Association Studies to Evaluate Polymorphisms Associated with the Risk of Differentiated Thyroid Carcinoma. Cancer epidemiology, biomarkers & prevention : a publication of the American Association for Cancer Research, cosponsored by the American Society of Preventive Oncology 25 (4):700-713. doi:10.1158/1055-9965.Epi-15-0652
16. Siraj AK, Ibrahim M, Al-Rasheed M, Abubaker J, Bu R, Siddiqui SU, Al-Dayel F, Al-Sanea O, Al-Nuaim A, Uddin S, Al-Kuraya K (2008) Polymorphisms of selected xenobiotic genes contribute to the development of papillary thyroid cancer susceptibility in Middle Eastern population. BMC medical genetics 9:61. doi:10.1186/1471-2350-9-61
17. Irmiakova AR, Kochetova OV, Gaĭnullina MK, Sivochalova OV, Viktorova TV (2012) [Association of polymorph variants of CYP1A2 and CYP1A1 genes with reproductive and thyroid diseases in female workers of petrochemical industry]. Meditsina truda i promyshlennaia ekologiia (5):41-48

18. Bufalo NE, Leite JL, Guilhen AC, Morari EC, Granja F, Assumpcao LV, Ward LS (2006) Smoking and susceptibility to thyroid cancer: an inverse association with CYP1A1 allelic variants. Endocrine-related cancer 13 (4):1185-1193. doi:10.1677/erc-06-0002

19. GallegosVargas J, SanchezRoldan J, RonquilloSanchez M, Carmona Aparicio L, FlorianoSanchez E, CardenasRodriguez N (2016) Gene Expression of CYP1A1 and its Possible Clinical Application in Thyroid Cancer Cases. Asian Pacific journal of cancer prevention : APJCP 17 (7):3477-3482

20. de Morais RM, Sobrinho AB, de Souza Silva CM, de Oliveira JR, da Silva ICR, de Toledo Nóbrega O (2018) The Role of the NIS (SLC5A5) Gene in Papillary Thyroid Cancer: A Systematic Review. International journal of endocrinology 2018:9128754. doi:10.1155/2018/9128754

21. Heinrich PC, Behrmann I, Müller-Newen G, Schaper F, Graeve L (1998) Interleukin-6-type cytokine signalling through the gp130/Jak/STAT pathway. The Biochemical journal 334 ( Pt 2) (Pt 2):297-314. doi:10.1042/bj3340297

22. Katoh M, Katoh M (2007) STAT3-induced WNT5A signaling loop in embryonic stem cells, adult normal tissues, chronic persistent inflammation, rheumatoid arthritis and cancer (Review). International journal of molecular medicine 19 (2):273-278

23. Hanavadi S, Martin TA, Watkins G, Mansel RE, Jiang WG (2006) Expression of interleukin 11 and its receptor and their prognostic value in human breast cancer. Annals of surgical oncology 13 (6):802-808. doi:10.1245/aso.2006.05.028

24. Goseki N, Koike M, Yoshida M (1992) Histopathologic characteristics of early stage esophageal carcinoma. A comparative study with gastric carcinoma. Cancer 69 (5):1088-1093. doi:10.1002/cncr.2820690503

25. Yamazumi K, Nakayama T, Kusaba T, Wen CY, Yoshizaki A, Yakata Y, Nagayasu T, Sekine I (2006) Expression of interleukin-11 and interleukin-11 receptor alpha in human colorectal adenocarcinoma; immunohistochemical analyses and correlation with clinicopathological factors. World journal of gastroenterology 12 (2):317-321. doi:10.3748/wjg.v12.i2.317

26. Vogelstein B, Fearon ER, Hamilton SR, Kern SE, Preisinger AC, Leppert M, Nakamura Y, White R, Smits AM, Bos JL (1988) Genetic alterations during colorectal-tumor development. The New England journal of medicine 319 (9):525-532. doi:10.1056/nejm198809013190901

27. Eun YG, Shin IH, Kim MJ, Chung JH, Song JY, Kwon KH (2012) Associations between promoter polymorphism -106A/G of interleukin-11 receptor alpha and papillary thyroid cancer in Korean population. Surgery 151 (2):323-329. doi:10.1016/j.surg.2011.07.014

28. Lin P, Guo YN, Shi L, Li XJ, Yang H, He Y, Li Q, Dang YW, Wei KL, Chen G (2019) Development of a prognostic index based on an immunogenomic landscape analysis of papillary thyroid cancer. Aging 11 (2):480-500. doi:10.18632/aging.101754

29. Zhong Z, Hu Z, Jiang Y, Sun R, Chen X, Chu H, Zeng M, Sun C (2016) Interleukin-11 promotes epithelial-mesenchymal transition in anaplastic thyroid carcinoma cells through PI3K/Akt/GSK3β signaling pathway activation. Oncotarget 7 (37):59652-59663. doi:10.18632/oncotarget.10831
30. Wang Y, Wei T, Xiong J, Chen P, Wang X, Zhang L, Gao L, Zhu J (2015) Association Between Genetic Polymorphisms in the Promoter Regions of Let-7 and Risk of Papillary Thyroid Carcinoma: A Case-Control Study. Medicine 94 (43):e1879. doi:10.1097/md.0000000000001879
31. Perdas E, Stawski R, Kaczka K, Zubrzycka M (2020) Analysis of Let-7 Family miRNA in Plasma as Potential Predictive Biomarkers of Diagnosis for Papillary Thyroid Cancer. Diagnostics (Basel, Switzerland) 10 (3). doi:10.3390/diagnostics10030130
32. Li M, Song Q, Li H, Lou Y, Wang L (2015) Circulating miR-25-3p and miR-451a May Be Potential Biomarkers for the Diagnosis of Papillary Thyroid Carcinoma. PloS one 10 (7):e0132403. doi:10.1371/journal.pone.0132403
33. Perdas E, Stawski R, Nowak D, Zubrzycka M (2016) The Role of miRNA in Papillary Thyroid Cancer in the Context of miRNA Let-7 Family. International journal of molecular sciences 17 (6). doi:10.3390/ijms17060909
34. Yuan L, Zhai L, Qian L, Huang D, Ding Y, Xiang H, Liu X, Thompson JW, Liu J, He YH, Chen XQ, Hu J, Kong QP, Tan M, Wang XF (2018) Switching off IMMP2L signaling drives senescence via simultaneous metabolic alteration and blockage of cell death. Cell research 28 (6):625-643. doi:10.1038/s41422-018-0043-5
35. Kloth M, Goering W, Ribarska T, Arsov C, Sorensen KD, Schulz WA (2012) The SNP rs6441224 influences transcriptional activity and prognostically relevant hypermethylation of RARRES1 in prostate cancer. International journal of cancer 131 (6):E897-904. doi:10.1002/ijc.27628
36. Yanatatsaneejit P, Chalermchai T, Kerekhanjanarong V, Shotelersuk K, Supiyaphun P, Mutirangura A, Sriuranpong V (2008) Promoter hypermethylation of CCNA1, RARRES1, and HRASLS3 in nasopharyngeal carcinoma. Oral oncology 44 (4):400-406. doi:10.1016/j.oraloncology.2007.05.008
37. Wilson CL, Sims AH, Howell A, Miller CJ, Clarke RB (2006) Effects of oestrogen on gene expression in epithelium and stroma of normal human breast tissue. Endocrine-related cancer 13 (2):617-628. doi:10.1677/erc.1.01165
38. Qu J, Liu L, Xu Q, Ren J, Xu Z, Dou H, Shen S, Hou Y, Mou Y, Wang T (2019) CARD9 prevents lung cancer development by suppressing the expansion of myeloid-derived suppressor cells and IDO production. International journal of cancer 145 (8):2225-2237. doi:10.1002/ijc.32355
39. Németh T, Futosi K, Weisinger J, Csorba K, Sitaru C, Ruland J, Mócsai A (2014) A8.25 CARD9 mediates autoantibody-induced autoimmune diseases by linking the SYK tyrosine kinase to CHEMOKINE production. Annals of the Rheumatic Diseases 73 (Suppl 1):A86-A86. doi:10.1136/annrheumdis-2013-205124.199
40. Liyanarachchi S, Gudmundsson J, Ferkingstad E, He H, Jonasson JG, Tragante V, Asselbergs FW, Xu L, Kiemeney LA, Netea-Maier RT, Mayordomo JI, Plantinga TS, Hjartarson H, Hrafnkelsson J, Sturgis EM, Brock

P, Nabhan F, Thorleifsson G, Ringel MD, Stefansson K, de la Chapelle A (2020) Assessing thyroid cancer risk using polygenic risk scores. Proceedings of the National Academy of Sciences of the United States of America 117 (11):5997-6002. doi:10.1073/pnas.1919976117
