# Supplementary material for: Genetic signature of differentiated thyroid carcinoma susceptibility: a machine learning approach
Source: Eur Thyroid J. 2022 Aug 17;11(5):e220058. doi: 10.1530/ETJ-22-0058 (PMC9513665; doi:10.1530/ETJ-22-0058)
Supplement: Supplementary Table S5: method of calculation of unweighted and weighted polygenic risk score (PRS). The unweighted PRS was built by summing the total number of risk alleles for each subject (attributing the value of 1 to each risk allele). In the weighted PRS, all the SNPs contribute to the total P [file supplementary_table_5.pdf]

Supplementary Table S5: method of calculation of unweighted and weighted polygenic risk score (PRS). The unweighted PRS was built by summing the total number of risk alleles for each subject (attributing the value of 1 to each risk allele). In the weighted PRS, all the SNPs contribute to the total PRS according to their association with the risk. It was built by assigning to each genotype the relative OR obtained in the GWAS. Then the ORs were multiplied.

	Genetic locus	1	2	3	4	5	6	7	10	11	12	13	14	15	16	17	18	19	20	21	
	Risk allele	T	C	T	T	T	A	A	T	T	C	C	A	G	T	A	A	G	G	A	
Subject																					
1		CC	CC	TT	CC	AG	CC	AA	AT	CC	CC	CT	GA	GA	GT	AA	AG	GG	AA	AA	
2		TT	TC	TT	CC	AA	CC	GA	AT	CC	CC	CC	GG	GG	GT	AG	AG	GA	AA	AT	
3		CC	CC	TC	CC	AA	CC	GG	AA	CC	CA	CC	GA	GG	TT	AA	GG	GG	AA	AA	
4		CT	TC	TT	CC	AA	CC	GA	AA	CT	CC	CC	GG	GA	GT	AG	GG	GA	AA	AA	
5		CT	TT	TT	CC	AA	CC	GA	AA	CC	CA	TT	GA	GG	GT	AA	AA	GG	AA	AT	
6		TT	TC	TC	CC	AA	CC	GG	AT	CC	CA	CT	GG	GG	GT	AA	GG	GG	AA	AA	
7		CT	TC	TC	CT	AA	CC	AA	AA	CC	CA	CC	GG	GG	GT	AG	AG	AA	AA	AA	
8		CT	TC	TT	CC	AG	CC	GA	AA	CC	CA	CC	GG	GA	GG	GG	AA	GG	AG	AA	
9		CT	TC	TC	CC	AA	CC	GA	AT	CC	CC	CC	GA	GA	GT	AA	AA	GG	AA	AA	
**Unweighted PRS**																					**PRS (is the summ)**
Subject																					
1		0	2	2	0	0	0	2	1	0	2	1	1	1	1	2	1	2	0	2	20
2		2	1	2	0	0	0	1	1	0	2	2	0	2	1	1	1	1	0	1	18
3		0	2	1	0	0	0	0	0	0	1	2	1	2	2	2	0	2	0	2	17
4		1	1	2	0	0	0	1	0	1	2	2	0	1	1	1	0	1	0	2	16
5		1	0	2	0	0	0	1	0	0	1	0	1	2	1	2	2	2	0	1	16
6		2	1	1	0	0	0	0	1	0	1	1	0	2	1	2	0	2	0	2	16
7		1	1	1	1	0	0	2	0	0	1	2	0	2	1	1	1	0	0	2	16
8		1	1	2	0	0	0	1	0	0	1	2	0	1	0	0	2	2	1	2	16
9		1	1	1	0	0	0	1	1	0	2	2	1	1	1	2	2	2	0	2	20
**Weighted PRS**																					
OR [a]		1.20	1.18	1.20	1.12	1.17	1.26	0.95	1.12	1.25	1.03	1.13	1.02	1.13	0.97	1.29	1.05	1.07	1.34	0.91	
OR [b]		1.31	1.43	1.24	1.28	1.41	1.67	1.17	1.40	1.60	1.37	1.56	1.21	1.24	1.21	1.31	1.27	1.31	1.95	1.91	
Subject																					**PRS (is the product)**
1		1	1.43	1.24	1	1	1	1.17	1.12	1	1.37	1.13	1.02	1.13	0.97	1.31	1.05	1.31	1	1.91	13.84
2		1.31	1.18	1.24	1	1	1	0.95	1.12	1	1.37	1.56	1	1.24	0.97	1.29	1.05	1.07	1	0.91	6.91
3		1	1.43	1.2	1	1	1	1	1	1	1.03	1.56	1.02	1.24	1.21	1.31	1	1.31	1	1.91	13.83
4		1.2	1.18	1.24	1	1	1	0.95	1	1.25	1.37	1.56	1	1.13	0.97	1.29	1	1.07	1	1.91	12.88
5		1.2	1	1.24	1	1	1	0.95	1	1	1.03	1	1.02	1.24	0.97	1.31	1.27	1.31	1	0.91	3.54
6		1.31	1.18	1.2	1	1	1	1	1.12	1	1.03	1.13	1	1.24	0.97	1.31	1	1.31	1	1.91	9.53
7		1.2	1.18	1.2	1.12	1	1	1.17	1	1	1.03	1.56	1	1.24	0.97	1.29	1.05	1	1	1.91	11.13
8		1.2	1.18	1.24	1	1	1	0.95	1	1	1.03	1.56	1	1.13	1	1	1.27	1.31	1.34	1.91	12.90
9		1.2	1.18	1.2	1	1	1	0.95	1.12	1	1.37	1.56	1.02	1.13	0.97	1.31	1.27	1.31	1	1.91	17.98

a= heterozygotes vs common homozygotes

b= rare homozygotes vs common homozygotes
